# Mesenchymal Stem Cells and Tissue Bioengineering Applications in Sheep as Ideal Model

**DOI:** 10.1155/2024/5176251

**Published:** 2024-10-18

**Authors:** Talita D'Paula Tavares Pereira Muniz, Mariana Correa Rossi, Vânia Maria de Vasconcelos Machado, Ana Liz Garcia Alves

**Affiliations:** ^1^Department of Veterinary Surgery and Animal Reproduction, School of Veterinary Medicine and Animal Science, São Paulo State University (UNESP), 18.618-681, Botucatu, Sao Paulo, Brazil; ^2^Materials Engineering Department (DEMa), São Carlos Federal University (UFSCar), 13.565-905, São Carlos, Sao Paulo, Brazil; ^3^Department of Veterinary Surgery and Animal Reproduction, Imaging Diagnostic Sector, School of Veterinary Medicine and Animal Science, São Paulo State University (UNESP), 18.618-681, Botucatu, Sao Paulo, Brazil

**Keywords:** biomaterial, osteogenesis, regenerative, scaffold

## Abstract

The most common technologies in tissue engineering include growth factor therapies; metal implants, such as titanium; 3D bioprinting; nanoimprinting for ceramic/polymer scaffolds; and cell therapies, such as mesenchymal stem cells (MSCs). Cell therapy is a promising alternative to organ grafts and transplants in the treatment of numerous musculoskeletal diseases. MSCs have increasingly been used in generative medicine due to their specialized self-renewal, immunomodulation, multiplication, and differentiation properties. To further expand the potential of these cells in tissue repair, significant efforts are currently dedicated to the production of biomaterials with desirable short- and long-term biophysical properties that can aid the differentiation and expansion of MSCs. Biomaterials support MSC differentiation by modulating their characteristics, such as composition, mechanical properties, porosity, and topography. This review aimed to describe recent MSC approaches, including those associated with biomaterials, from experimental, clinical, and preclinical studies with sheep models.

## 1. Introduction

Musculoskeletal disorders are currently among the main causes of disability, accounting for 25% of all surgical procedures in the United States [1]. These disorders can be caused by acute trauma, such as fractures or sports injuries; tissue degeneration, such as osteoarthritis and spinal stenosis; genetic conditions, such as muscular dystrophy; and autoimmune diseases, such as rheumatoid arthritis. Moreover, fewer therapies are available for chronic musculoskeletal diseases [2].

Bone grafts, such as autografts and allografts, have been commonly used to treat degenerative or traumatic bone injuries. Nevertheless, bone grafts have had limited success in clinical practice. These procedures are also expensive and associated with complications, such as risk of infection, graft rejection, and poor osteoconductivity. Thus, alternative clinical techniques and materials that are safe, economical, and reliable are required [3]. Consequently, healthcare professionals and researchers have dedicated their efforts to the fields of tissue engineering and regenerative medicine to provide effective and appropriate solutions to the challenges presented by musculoskeletal diseases [4].

When analyzing potential musculoskeletal injuries and regenerative therapies, sheep are usually employed as large animal models [5]. Sheep are excellent models for the analysis of potential biological changes related to the successful or unsuccessful use of titanium alloys [6]. Adult sheep are ideal for translational research, tissue engineering, and biomaterial studies as they are docile, easy to handle, and have anatomical structural similarities with human bones [7, 8].

In recent decades, the development of bone tissue structures associated with orthobiologics has been the primary focus of tissue engineering research [9] to ultimately identify more effective and safe alternatives. Tissue engineering based on biomaterials uses many different types of biocompatible cell-seeded structures to enhance cell viability, growth potential, and differentiation in distinctive transplanted stem cell lineages [10]. The combination of various biomaterials with stem cells better simulates the in vivo environment and is a significant tool for controlling the viability and fate of stem cells, favoring cell-to-cell and cell-to-matrix interactions [11]. Furthermore, other strategies in orthopedic regenerative medicine have not been more promising than mesenchymal stem cell (MSC) therapies for musculoskeletal diseases. Nevertheless, these therapies must be assessed using appropriate models and be standardized before advancing to the preclinical stage [12].

## 2. Methods

Herein, a comprehensive review of scientific articles and clinical trials on the therapeutic applications of MSCs in sheep models is presented. The keywords were “mesenchymal stem cells,” “sheep,” “tissue engineering,” “regenerative medicine,” and “cell-based therapy.” PubMed/Medline, SciELO, and Web of Science were used for the literature search. This review was based on studies that met the following eligibility criterion: studies involving the use of MSCs in sheep models as a therapy in the preclinical and clinical settings.

MSCs: origins, main applications, and associations with tissue bioengineering.

### 2.1. Characteristics of a Cell That is Considered a Stem Cell

Stem cells have been considered representative cells since their first isolation in 1970 [13]. However, the minimum requirements for defining MSCs were standardized by the Society for Cell Therapy in 2006 [14]. These cells must be adherent to plastic under standard culture conditions; must be able to differentiate into osteoblasts, chondrocytes, and adipocytes in vitro; and must express the surface antigens cluster of differentiation markers (CD)105, CD73, and CD90, in the absence of CD45, CD34, CD14, or CD11b, human leukocyte antigen—DR isotype (HLA-DR), CD19, or those specific to human cells [15]. However, the proposed CD expression is variable in the most diverse stem cell sources [16].

The German scientist, Ersnt Haeckel, introduced the term “stammzelle,” or stem cell, in the 19th century, which was defined as a unicellular organism from which all multicellular organisms are generated. The term “stem cell,” as known today, was first used in studies on the regeneration of hematopoietic tissues. Another German scientist, Pappenheim, was the first to mention the term “stammzelle” in his manuscripts on hematopoietic tissue. However, the Russian-American scientist, Alexander Maximow, was the first to use the term “stem cell” to define populations of cells from which all hematopoietic lineages are generated through their local influence on bone marrow stroma.

In the 20th century, adult stem cells were defined as a population of cells that can self-renew and result in differentiated cells of particular tissues. All adult tissues contain a population of stem cells, which are found in specific microenvironments and form progenitor cells that eventually differentiate [17]. Thereafter, research that aimed to learn more about the biology of stem cells became increasingly common, mainly due to the potential of stem cells to improve healthcare through regenerative medicine cell therapy [18].

When a second class of stem cells was discovered in the bone marrow, scientists focused on the potential of these cells to form bone tissue and provide hematopoietic support in the stroma [19]. Zuk et al. [20], in 2002, were the first to isolate adipose tissue-derived stem cells (ADSCs) from humans.

Stem cells are broadly defined as cells that can replace injured tissues owing to properties guided by their microenvironment [21]. Caplan [22] was the first to use the term “mesenchymal stem cell” in 1991. He hypothesized that the bone marrow stem cell was the adult cell of an embryonic MSC and could form cartilage, bone, tendon, ligament, medullary stroma, adipose tissue, dermis, muscle, and connective tissue throughout development [22]. This hypothesis became practical 8 years later owing to the publication by Pittenger et al. [23]. According to these investigators, human bone marrow MSCs could be induced to differentiate into osteogenic, adipogenic, and chondrogenic lineages under specific cell culture conditions. Therefore, adult MSCs could be promising therapeutic agents for the repair of several tissues, not just bone tissues [22].

### 2.2. Characterization of MSCs

Stem cells can be categorized into two types: embryonic stem cells (ESCs) and non-ESCs, also called somatic or adult stem cells [24]. Regarding ESCs, Chen et al. [25] summarize the main transcription factors, signaling pathways, and regulation of self-renewal of such cells. However, there are several ethical dilemmas involved in the use of ESCs, involving two moral principles that are not possible to be met simultaneously in healthcare: the duty to prevent and/or alleviate suffering and the duty to respect the value of human life, since the embryos would have the potential to become human beings. In addition to several other potential impacts that are normally unrelated and that were explained by Assen et al. [26]. There are several other sources of stem cells that can be analyzed and used.

Adult stem cells can only differentiate into cell types within a specific lineage [27]. Furthermore, Lanzillotti et al. [28] summarized the roles of long noncoding RNAs (IncRNAs) with microRNAs (miRNAs) as key regulators during osteogenic differentiation of bone marrow-derived MSCs. Such clarifications allowed better knowledge about the regulation of gene expression related to bone development, bone homeostasis as well as bone regeneration.

Induced pluripotent stem cells (iPSCs) are another type of adult stem cells that can differentiate into the three germ layers (ectoderm, mesoderm, and endoderm). However, several ethical issues and the potential for tumor development owing to the use of these induced cells [29] led to the search for another type of stem cells, MSCs [30].

**Figure 1:**
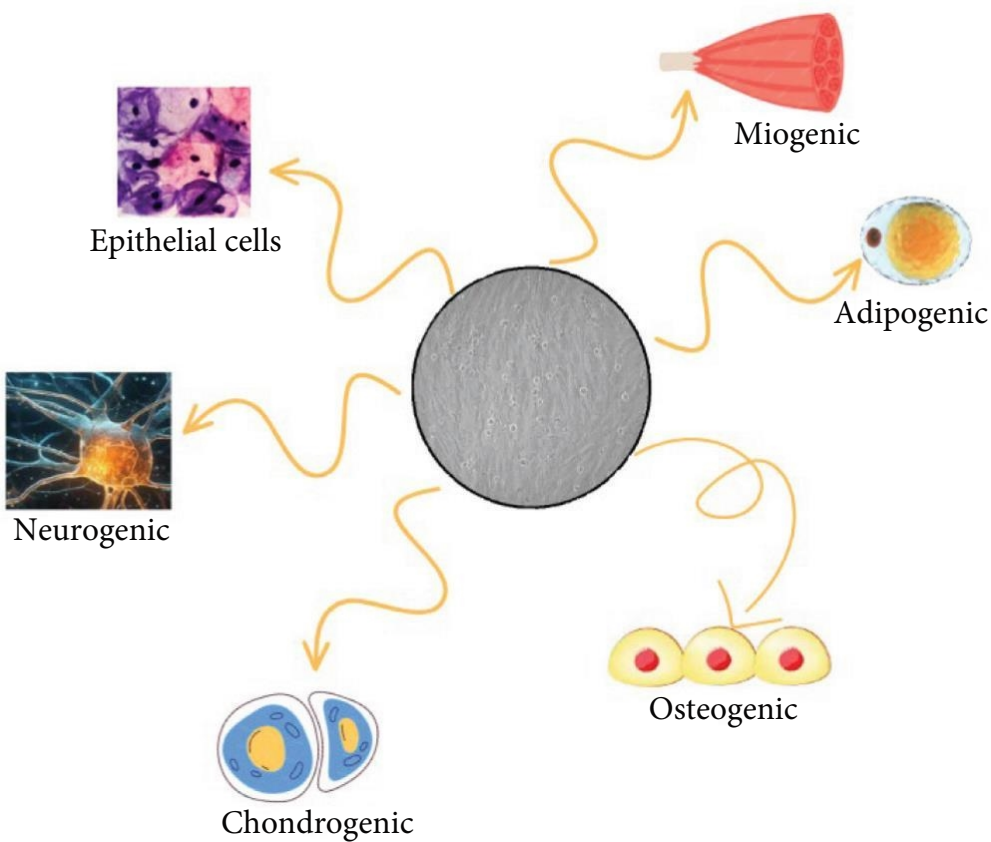
Main MSCs differentiation lineages. MSCs, mesenchymal stem cells.

MSCs are multipotent cells that can generate diverse lineages (Figure 1) according to the environmental signals received [31]. The development of MSCs depends on the extrinsic microenvironment; thus, when used in tissue injuries, responses vary depending on the type of tissue [32]. MSCs are cells that easily adhere to plastic surfaces, have high expansion capacity on larger scales, high differentiation capacity, and have no side effects, as established by several clinical trials [30].

As MSCs lack costimulatory molecules, such as CD80, CD86, and CD40, which are necessary for the activation of T cells, they are immunologically desirable. MSCs also have a low expression rate of class II major histocompatibility complexes (MHCs); combined with their immunomodulatory property and differentiation potential, MSCs are essential in the treatment of the most diverse autoimmune diseases [33].

MSCs are isolated from the umbilical cord, placenta, bone marrow, liver, chorionic tissues, and adipose tissues. The adipose tissue is the most used as it is more easily accessed. However, such acquisitions require relatively invasive surgical procedures to obtain them [34–36]. Another possible related source is Wharton's umbilical cord jelly (WJ-CTMs), which has unique properties, such as ease of isolation and culturing, availability in different tissues, ability to self-regenerate and differentiate into different cell lineages, in addition to the absence of ethical issues related to its use and not requiring surgical procedures to obtain it [37, 38].

Bone marrow MSCs are a group of heterogeneous, multipotent cells with significant immunomodulatory capacity, being widely explored among the various therapies in immunological disorders and with bioengineering [39, 40]. Although the differentiation and hierarchies of hematopoietic stem cells have long been well understood, those of bone marrow-derived mesenchymal stem cells (BMSCs) are less well defined [41]. However, they can be acquired in a practical way, and their in vitro cultivation allows easy and rapid amplification, in addition to known genetic stability. There are no related ethical issues, since they can also be transplanted autologously [42–44].

Once obtained, there are several methods for isolating MSCs from tissues, and each method has advantages and disadvantages, depending on the type of tissue to be processed and the specific objectives for its use. Centrifugal separation is a quick, simple, and less invasive process that uses cell density as a separation criterion. However, this may result in a lower MSC isolation rate. Another possible way is through the enzymatic digestion method, which is more complex, invasive and uses enzymes to degrade the tissue into fragments that release target cells. It is generally more effective for obtaining greater quantities of MSCs [45].

After collection and culture, characterization of MSCs is performed, which begins with their attachment to cell culture plates. As the morphology of MSCs is similar to that of fibroblasts and they can differentiate into diverse mesenchymal lineages, the rate of proliferation and colony formation is high [46]. These cells are also multipotent, adherent, and represent progenitor cells with diverse differentiation potential [14].

In order to identify and characterize MSCs based on their cell surface markers, immunophenotyping can be used. Markers are proteins expressed on cell surfaces that can be detected by specific antibodies using flow cytometry to identify and quantify MSCs and evaluate the expression of various related surface markers [47]. Several positive and negative markers ensure that the isolated cells are MSCs. These markers include glycophorin-A, an erythroid lineage marker; CD11b, an immune cell marker; CD31, a producer of endothelial and hematopoietic cells; CD34, a primitive hematopoietic stem cell marker; CD45, a marker of all hematopoietic cells; and CD117, a hematopoietic stem cell marker [48]. Some markers are present in some species and not in others; thus, the following positive markers are also used: Stro-1, CD10, CD13, CD29, CD44, CD73, CD90/Thy-1, CD105, CD106, CD271/NGFR, CD309/Flk-1, and Sca-1. Among them, Stro-1 is the most used, as it does not form colonies in cells negative for Stro-1 [49].

MSCs can be renewed without differentiation. These cells express genetic markers of ESCs, OCT-4, SOX-2, and REX-1, which are involved in the repression of differentiation genes. Leukemia inhibitory factors and fibroblast, hepatocyte, epidermal, platelet-derived growth factors, and beta-catenin are also present. Beta-catenin is an extracellular matrix (ECM) protein that anchors cells in place and plays a role in stem cell self-renewal [50]. The differentiation process is induced through the administration of transforming growth factor beta (TGF-*β*), bone morphogenetic protein (BMP), growth and differentiation factors, and Wnt ligands for chondrogenesis, tenogenesis, and osteogenesis. Peroxisome proliferator-activated receptor gamma (PPARgamma) and Notch 1 are used for adipogenesis and myogenesis, respectively. Biomaterials are also used to stimulate the differentiation of cells into a specific lineage [51].

MSCs have immunomodulatory properties and can attenuate tissue injury, inhibit fibrotic remodeling and apoptosis, promote angiogenesis, stimulate the recruitment and proliferation of stem cells, and reduce oxidative stress [52]. MSCs can also modulate the immune response induced by B cells, T cells, and natural killer (NK) cells through the secretion of paracrine factors and cell-to-cell contact [33]. Notably, MSCs induce cell survival and angiogenesis, and prevent tissue fibrosis [53]. Important mediators related to this remodeling are metalloproteinases (MMPs) and tissue inhibitors of MMPs (TIMPs), which protect implanted cells [54].

Due to their properties, especially their ability to self-regenerate, immunomodulation and differentiate into several cell lineages, MSCs have become a promising tool in efficient treatment strategies.

### 2.3. Clinical Uses of MSCs

**Figure 2:**
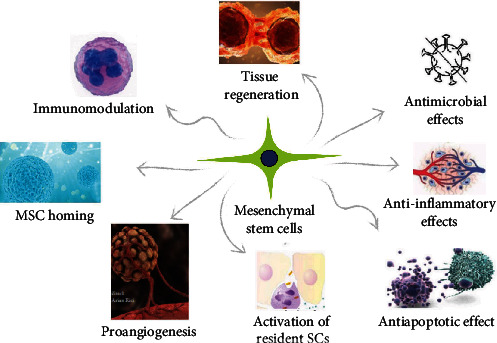
Scheme presenting different mechanisms related to MSCs and their therapeutic potential. MSCs, mesenchymal stem cells.

MSCs have diverse mechanisms to fulfill their therapeutic potential (Figure 2), such as anti-inflammatory action, antimicrobial effects, antiapoptotic activity, immunomodulation, proangiogenesis, resident endogenous stem cell activation, and tissue repair and regeneration [55].

Recently, numerous in vivo and in vitro studies have provided new insights into various regenerative medicine applications of adult MSCs in trauma and tissue injuries. MSCs have also been suggested to promote the recovery and remodeling of both the stroma and parenchyma in response to stress and traumatic events [56]. These cells have been used in numerous trials to treat neurological [57], renal [58], hepatic [59], cardiovascular [60], pulmonary [61], and musculoskeletal [62, 63] diseases.

Sports injuries, accidental trauma, aging, and degenerative conditions, such as osteoarthritis, can cause extensive damage to articular cartilage. Current therapies are used to alleviate clinical signs, reduce pain, and control inflammation. However, these therapies do not control the progressive degeneration of joint tissues [64].

Mazziotta et al. [65] highlighted the main studies involving circular RNAs (circRNAs), which regulate the self-renewal, proliferation, and osteogenic differentiation of human MSCs (hMSCs). hMSCs have important biological properties with an important role in both natural bone repair and related bone tissue bioengineering approaches. For example, the *in vitro* study by Ji et al. [66] with BMSCs isolated from patients with senile osteoporosis and deregulation of RUNX2 in several circRNA/miRNA axes. Dysregulated expression of RUNX2 is frequently associated with the development of osteoarticular diseases.

Osteosarcoma, an aggressive and primary high-grade tumor, is located mainly at the ends of long bones and close to the growth plates, mainly in the distal femur, proximal tibia, and proximal humerus. The most common clinical signs involve reduced joint movement, local pain, and edema around the tumor mass, varying according to its location and growth rate [67]. It is considered a disease of cellular differentiation derived from the transformation of MSCs. Studies have, therefore, explored the role of miRNAs in the regulation between MSCs and osteosarcoma-derived cells using MSC-derived exosomes. Depending on the type of miRNAs, there are tumor-promoting and tumor-suppressing organelles, suggesting modulation of the behavior of cancer cells in osteosarcoma. That is, microenvironmental signals from surrounding MSCs are the main contributors to the development of osteosarcoma. A better understanding of these pathways and the interactions of embryonic markers and the tumor microenvironment can be a promising therapeutic strategy to overcome chemoresistance and improve the prognosis of affected patients [68].

In a phase I/II randomized controlled clinical trial, success was achieved with the use of repeated doses of umbilical cord-derived MSC in knee osteoarthritis processes approximately 12 months after the start of treatment. A comparison was made with a single dose of MSC and hyaluronic acid. This concerns its paracrine action and subsequent effects on the cartilage regeneration process [69].

In other cases of bone fracture repair, defects secondary to trauma and posttumor resection or postdebridement continue to be a major problem in clinical medicine, generating high consequential costs [70, 71]. A promising alternative is the combination of porous and osteoconductive scaffolds seeded with MSCs, mainly BMSCs [72].

Clinical cases of fracture pseudoarthrosis are still considered significant clinical problems in orthopedics. When adequate fracture healing does not occur, usually between 6 and 8 months later, there is nonunion or delay, resulting in increased medical expenses and prolonged hospitalization. Re et al. [73] listed the main clinical trials that addressed the use of scaffolds with or without MSCs to repair bone defects. Among the selected studies, six used scaffolds with MSC cells, and three used only the scaffolds. Most of the scaffolds were composed only of calcium phosphate ceramics, such as *β*-tricalcium phosphate (TCP) (two clinical trials), bioceramic granules of biphasic calcium phosphate (three clinical trials), and inorganic bovine bone (two clinical trials), while bone marrow was the main source of MSCs (five clinical trials). Among the diseases analyzed, Blanco et al. [74] investigated degenerative disc disease in lumbar vertebrae using beta-TCP scaffolds associated with BMSCs. They observed significant improvement in the first year after surgery, and approximately 80% of patients achieved lumbar fusion within 5 years, with no adverse effects after implantation.

The great potential and availability of MSCs allow for their various clinical applications in the treatment of many incurable and challenging diseases.

### 2.4. MSCs in Orthopedics in a Sheep Model

**Figure 3:**
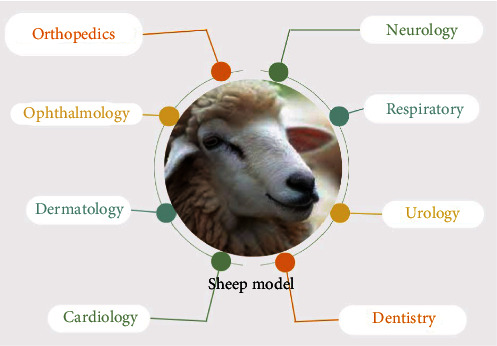
Clinical applications of MSCs evaluated using sheep models. MSCs, mesenchymal stem cells.

The choice of an animal model is mainly influenced by practical aspects, such as ethics, costs, and ability to accommodate the materials and competent personnel. Currently, obtaining ethical permission for the use of sheep or goats is easier than obtaining such permission for the use of dogs and horses. Surgical limitations, such as the animal's tolerance to anesthesia and postsurgical recovery protocols, also influence the choice of the animal model [75]. The sheep model (Figure 3) is economical and particularly easy to handle and anesthetize [76].

Adult sheep are ideal for translational research, tissue engineering, and biomaterial studies as they are docile and easy to handle and have anatomical structural similarities with human bones and phylogenetic relatedness with humans [7, 8, 77]. The sheep model is well documented. The most frequent natural cartilage defects in sheep knees occur on the axial aspect of the medial tibial condyle (MTC) and medial femoral condyle (MFC) [78].

Among the main limitations related to the use of sheep as animal models in research, the following stand out: in relation to housing these animals, which requires more space and is generally not widely available; about molecular tests and their byproducts, where their commercial availability is more limited when compared to rodents [79]; in studies that aim to analyze the effectiveness of medications according to physiological characteristics, because small ruminants have three prestomachs and one true stomach, the evaluation dynamics differ to another species [80].

Ribitsch et al. [81] also summarized the main disadvantages of sheep as an experimental animal model, such as the occurrence of different breeds, generally not bred specifically for research; need for specific facilities required for accommodation, surgery, diagnostic imaging, necropsy and technical skills in their management; need to control weight gain, especially postoperatively; costs arising from feeding and handling, in addition to resulting ethical concerns, but to a lesser extent when compared to companion animals. However, it is known that the related disadvantages can be easily resolved in practice [79].

Table 1 shows some studies related to the main clinical applications of MSCs using sheep as animal models.

Based on most of the clinical results reported to date, MSC therapy is viable, beneficial, and safe as it improves functionality, reduces pain, and promotes the regeneration of injured cartilage and in skin wound tissue repair.

### 2.5. Scaffolds and Orthobiologic Therapies

At the beginning of the bone tissue repair process, the recruited MSCs, osteoprogenitor cells, and osteoblasts must attach to an osteoconductive scaffold to ensure cell activities in tissue repair proceed in an appropriate manner. When this support, whether natural or artificial, is absent, bone regeneration may be compromised, and fibrous tissue may fill the defect with a low-quality cellular matrix before the bone tissue [89].

Natural bone consists of an ECM composed mainly of collagen fibers, hydroxyapatite, interstitial fluid, and cells [90]. Approximately 10%–30% of bone is composed of a hard, porous outer layer, called cortical bone. The remaining 70%–90% is composed of a porous inner layer, called cancellous bone [91]. Combining the natural properties of bone with implants is a major challenge in bone tissue repair. The greater the similarity, the greater the chance of the body's acceptance of the structure and new tissue growth [92].

Technological advances have led to the development of biomaterials that reconstruct injured bone. These bioactive materials can cause controlled actions and reactions in the biological environment. However, biomaterials alone are only guides for bone tissue, not osteoinductive agents; thus, immature cells are not recruited or stimulated to differentiate into osteogenic cells. Tissue engineering constructs composed of cells, bioactive structures, and bioactive factors are necessary to guarantee structural and functional integrity in complex musculoskeletal disorders [93].

Scaffolds are three-dimensional constructs designed and intended to recreate the ECM. Scaffolds promote the regeneration of functional bone and allow stem cells to be seeded in controlled and protected spaces for their self-renewal and survival. These scaffolds guide the healing process, promote the differentiation of progenitor cells, and emulate the extracellular environment as much as possible while also providing biomechanical support [94]. Several types of scaffolds have already been used for bone tissue engineering and synthesized with inorganic and organic materials [72]. This method is favorable because it produces a scaffold with a homogeneous distribution that can incorporate an identical and well-defined dose [95].

To achieve ideal integration and adequate stability, the scaffolds must be biocompatible, depicted by their ability to stimulate minimal inflammatory and immunological response; be biodegradable, can be completely replaced over time by autologous tissue; mimic the ECM as much as possible; and facilitate mineral deposition and provide an adequate physical structure for bone growth in and through it (i.e., osteoconduction) [96]. Osteoconductive materials are mainly effective with partially differentiated cells, such as osteoblasts and preosteoblasts, although they do not induce osteogenic differentiation of bone progenitor cells and MSC [97]. On the other hand, these osteoinductive biomaterials can recruit progenitor cells and stimulate osteoblastic differentiation, which allows new bone tissue formation. Therefore, biomaterials are not only load-bearing materials for tissue reconstruction but are also significant differentiation inducers [98].

Tissue engineering implants enriched with stem cells or osteogenic factors, such as BMPs, are osteoinductive materials [99]. Besides these osteoinductive properties, complete osseointegration of the structure into the tested bone tissue is necessary, which directly depends on adequate vascular support stimulated by the implant (angioinduction). The implant's stable vascularization (angioconduction) and a vascular supply to its central part maintain bioactive function and avoid necrosis, presenting a significant risk, especially in large implant areas [100].

Growth and porosity stimulation provide greater biocompatibility between tissue and implant (scaffold) [101]. Magnesium is excellent for the development of porous materials. Moreover, magnesium can be used as a spacer as it provides porosity in metals and alloys and contributes to the osteoinductive process [102]. Thus, pore size optimization is crucial during implant construction and for cell differentiation, proliferation, and migration, as it enables ideal structure integration and adequate bone tissue engineering [103].

Several types of implants associated with MSCs and used for bone reconstruction are derived from organic and inorganic sources. The materials can be metallic, such as titanium, stainless steel, cobalt chrome, or tantalum alloys, with or without surface treatments to promote osseointegration; ceramics, such as aluminum, zirconium, or porous ceramic; or polymers, such as polyethylene, acrylic resins, polyurethane, and polymethyl methacrylate, polymeric compounds, such as polyetheretherketone, and synthetic biodegradable polymers, such as polylactic acid and polyglycolic acid; and combinations of two or more [104].

Wang et al. [105] developed a porous titanium alloy rod with a diamond crystal structure and implanted in vivo in sheep for 6 months. The rods induced adequate osteoconduction and appropriate growth of new bone tissue. Furthermore, titanium implants showed greater bone volume in microtomography, greater bone growth, and enhanced mechanical properties, thereby becoming a promising treatment for femoral head osteonecrosis.

Yang et al. [106] observed that structures optimized in their microenvironment relevantly modulate the fate of MSCs and promote cell migration, proliferation, and chondrogenesis. This strategy better-accomplished regeneration in sheep using scaffolds with biomechanical and antiapoptotic properties.

Dreyer et al. [107] tested four 10 × 12 mm titanium implants inserted bilaterally into the distal femurs at 2-mm intervals in eight sheep. Autologous MSCs seeded on hydroxyapatite granules were added, with distinct releases of vascular endothelial growth factors (VEGFs) per day. MSCs and VEGFs allowed significant bone regeneration, with bone properties equal to the allograft and no systemic increase in osteogenic markers or VEGF without visible side effects.

In the case of skeletal bone defects resulting from tumor resection, trauma, or diseases, such as osteoporosis, they require immediate clinical intervention in order to promote good healing and restoration of function. There is the production of biomaterials that serve as support for association with orthobiological therapies that promote adequate cell distribution, provide structural support and stimulate bone repair with an adequate matrix. Thus, Markides et al. [108] analyzed viability, biodistribution, and exogenous infiltration by creating a critical size defect in sheep 2 and 7 days postimplantation of STRO-4 autologous mesenchymal stromal cells within a bone ECM gel of origin. Natural. A reduction in the number of therapeutic cells was observed after implantation at the repair site but without concomitant inflammatory blood markers. Therefore, an essential factor when dealing with orthobiological therapies is understanding the optimal therapeutic dose of cells and understanding their fate after implantation.

Regarding large bone defects to be repaired, it is known that the ideal solution is to associate a scaffold with orthobiological therapies, promising therapies for bone regeneration. Thus, Szaÿaj et al. [109] evaluated the effect of fibroblast growth factor 2 (FGF-2) and BMP-2 on the osteogenic potential of bone marrow MSCs (BM-MSCs) seeded on a polycaprolactone (PCL)/hydroxyapatite (HAP)/*β*-TCP scaffold coated with nano-hydroxyapatite (nHAP) in vitro and analyzed in vivo in sheep. Biocompatibility of the scaffold produced was observed through the expression of early collagen (type I collagen) and late osteogenic proteins (osteocalcin), in addition to the absence of high levels of pro-inflammatory cytokines.

Several therapeutic approaches are currently being tested aimed at repairing osteochondral tissue, such as autologous osteochondral transplantation or through the production of microfractures. However, none of the approaches achieved repair of the osteochondral unit. Thus, Sanjurjo-Rodriguez et al. [110] isolated and characterized BM-MSCs from sheep in order to evaluate their capacity in osteochondral repair with the aid of a ÿ-TCP and type I collagen scaffold. It was observed that with scaffolds seeded with BM-MSCs and Col I they can adequate fibrocartilage and hyaline cartilage tissue is formed in osteochondral cartilaginous repair.

In *in vitro* studies, De Mori et al. [111] evaluated 3D-printed silica scaffolds with different pore sizes, 200 µm (SC-200) or 500 µm (SC-500), to analyze chondrogenesis in association with BM-MSCs. The following were analyzed: cell attachment, viability, proliferation, morphology, expression of chondrogenic genes, deposition of aggrecan, collagen types I and II, and quantification of sulfated glycosaminoglycans. It was found that the scaffold allows cell fixation and proliferation with upregulation of chondrogenic markers and deposition of extracellular cartilage matrix with type II collagen and aggrecan. Furthermore, pore sizes of 200 µm are ideal for promoting chondrogenic differentiation of BM-MSCs.

Orthobiological therapies, such as MSCs, in association with biomaterials, known as scaffolds, are undoubtedly the great hope for the treatment of many diseases and conditions. As in many adult tissues and do not raise ethical issues, they have excellent advantages over other techniques that have not considered the quality of the matrix produced and over other sources like ESCs. Whether in vivo or in vitro studies, there is no doubt that the cells can migrate and make homing to the injured tissues, which seems to be a promising tool in regenerative medicine.

### 2.6. Future Prospects of Tissue Engineering With MSCs

Wolter and Meyer [112] were the first to use the term “tissue engineering” in 1980. Thereafter, Kaiser [113] revealed a new therapeutic strategy encompassing the regeneration or repair of an injured tissue or organ. This concept evolved into regenerative medicine, which is a medicine approach comprising several techniques that aim to regenerate damaged cells, tissues, or organs by stimulating the body's own repair mechanism [114].

Once damaged, articular cartilage tends to suffer continuous degradation due to its location and limited innate healing potential. Furthermore, considering the limitations of current surgical techniques, the incorporation of stem cells in regenerative medical therapy has been extensively studied for adequate cartilage rehabilitation [115].

Stem cells are cells that can self-regenerate and differentiate into other types of cells. Stem cell therapies represent a new regenerative medicine approach to sports injuries. These therapies enable intervention at the physiological level through cellular differentiation and at the molecular level through chemotaxis of various cytokines, hormones, and growth factors that allow tissue repair [116]. MSCs are widely used as a potential cell-based therapy for the treatment of sports-related injuries [117].

The potential of stem cells has created great expectations and led to an increase in clinical translational investments. However, concerns about the marketing and sale of stem cell products have emerged, in addition to their use in unproven treatments that could harm patients [118]. Therefore, the International Society for Cell and Gene Therapy (ISCT) published a document to inform professionals and patients about unproven cell-based therapies [119]. In 2016, the International Society for Stem Cell Research (ISSCR) updated this document to promote efficient, appropriate, and sustainable stem cell research and establish standards for clinical research conduct. Some of the recommendations are described below: the release of clinical trials must be supported by scientific evidence; risks must be identified and minimized; and stem cell-based therapies must be safe and effective and must aim to outdo existing therapies. Thus, veterinary medicine must consider the same concerns for the clinical application of MSC-based products [120].

Most clinical assays with cell therapies for musculoskeletal diseases comprise cases with no adequate control. Moreover, assessment of the improvement of stem cell tissue regeneration would be easier with new noninvasive tissue quality metric approaches that are biochemical or imaging-based. These technologies would allow real-time monitoring of scar tissue compared to that of postdeath histologic assessment in animal models [2].

Regardless of source, one of the biggest challenges in the clinical application of stem cell-based bioengineering techniques is large-scale in vitro expansion before transplantation to obtain adequate concentrations of stem cells. Usually, millions to hundreds of millions of stem cells are needed per patient. Scaffolds based on porous biomaterials can overcome these limitations, supporting cell adherence, survival, proliferation, and differentiation [121].

Emerging biomaterials placed in musculoskeletal injuries can recruit endogenous stem cells to the scaffold, where incorporated bioactive growth factors can lead to differentiation that is appropriate to the site [122]. Clarification of these complex interactions between the cell components of bone will increase the therapeutic uses of MSCs associated with tissue engineering for better bone repair. Automation and standardization would also improve bone tissue engineering and increase the likelihood of effective and economically viable technologies [2].

## 3. Final Considerations

An intense search is ongoing to enable the development of new complementary materials for biological products, especially with deeper multidisciplinary integration. However, gaps between clinical applications and mechanical studies still exist, in addition to potential adverse effects and misdirected indications, which prevent the progress and optimization of clinical treatments for various musculoskeletal diseases. Furthermore, appropriate clinical practices in human and veterinary medicine depend on the choice of appropriate animal models that simulate tissue repair processes as much as possible, ultimately improving research on bone consolidation mechanisms and promoting the development of economic and effective therapeutic methods in the short and long terms.

## Figures and Tables

**Table 1:** Studies related to the main clinical applications of MSCs using sheep as animal models.

*N* sample	Main findings	Reference
9 sheep—2 osteochondral lesions at the medial femoral condyles in hind legs	Confirmed the efficacy of cartilage regeneration from bone marrow MSCs (BM-MSCs) and MSCs differentiated into chondrogenic lineage in sheep models after 12 months.	Marquass et al. [82]

16 sheep—3 groups: surgically induced osteoarthritis	Intra-articular injection of a single dose of BMSC either chondrogenically induced or not, could retard the progression of osteoarthritis in a sheep model, but the induced cells indicated better results especially in meniscus regeneration.	Al Faqeh et al. [83]

16 sheep—2 groups: induced monoarthritis by administration of bovine type II collagen in left hock joint	Reductions in lameness, pain, joint swelling and in cartilage erosions; activation of synovial stromal cells and angiogenesis, and infiltration of synovial tissues with CD4 lymphocytes and CD14 monocytes or macrophages have also been observed.	Abdalmula et al. [84]

30 sheep—28 surgically induced OA on right knee	IA injection of allogeneic AD-MSCs combined with HA could efficiently block OA progression and promote cartilage regeneration and allogeneic AD-MSCs might survive at least 14 weeks after IA injection.	Feng et al. [85]

18 sheep—3 groups: OA induced on right knee joint	Bone marrow cells showed therapeutic efficacy in a sheep model of OA. Also demonstrated the inhibition of PGE_2_, TNF-*α* and TGF-*y* levels in synovial fluid, regulation of MMP-13 in chondrocytes and increased expression levels of aggrecan and COL2A1.	Song et al. [86]

20 sheep—4 groups: OA induced via unilateral medial meniscectomy	The transplantation of BMC-HA provided the best effects in supporting regenerative processes in cartilage, meniscus, and synovium and at less extent in bone. On the whole, both MSC and BMC combined with HA reduced inflammation and contributed to switch off fibrotic and hypertrophic processes.	Desando et al. [87]

6 sheep—clinical, histopathological and molecular analysis approaches to evaluate the action of allogeneic MSCs 15 and 42 days after injury	Wounds treated with MSCs exhibited a greater degree of re-epithelialization, proliferation, neovascularization, and increased contraction after 15 days compared to control wounds. At day 42, wounds treated with MSCs also had more mature and dense skin appendages compared to the control.	Martinello et al. [88]

Abbreviations: AD-MSCs, adipose tissue-derived mesenchymal stem cells; BM-MSCs, bone marrow MSCs; HA, hyaluronic acid; IA, intra-articular; MFC, medial femoral condyle; MMP, metalloproteinase; MSC, mesenchymal stem cell; OA, osteoarthritis; PGE, prostaglandin E; TGF-*γ*, transforming growth factor gamma; TNF-*α*, necrosis tumoral factor alpha.

## Data Availability

The research data supporting this systematic review are from previously reported studies and datasets, which have been cited. The processed data are available from the corresponding author upon request.
